# Impact of the use of the ultra-portable digital x-ray with CAD4TB for active case finding for tuberculosis in Nigeria

**DOI:** 10.3389/fdgth.2025.1559203

**Published:** 2025-06-30

**Authors:** Abiola Alege, Sani Useni, Austin Ihesie, Rupert Eneogu, Aderonke Agbaje, Bethrand Odume, Debby Nongo, Eze Chukwu, Chidubem Ogbudebe, Omosalewa Oyelaran, Chris Anyomi, Samuel Akingbesote, Zhi Zhen Qin, Rachael Barrett, Obioma Chijioke-Akaniro, Chukwuma Anyaike, Atana Ewa

**Affiliations:** ^1^ Programs, Society for Family Health, Abuja, Nigeria; ^2^ Programs, KNCV Nigeria, Abuja, Nigeria; ^3^ HIV AIDS & TB Office, United States Agency for International Development, Abuja, Nigeria; ^4^ National Tuberculosis Research Task Team, Abuja, Nigeria; ^5^ Programs, Institute of Human Virology, Abuja, Nigeria; ^6^ Programs, Center for Integrated Health Program, Abuja, Nigeria; ^7^ Digital Health, Stop TB Partnership, Geneva, Switzerland; ^8^ Department of Public Health, Federal Ministry of Health, Abuja, Nigeria; ^9^ Department of Paediatrics, University of Calabar/University of Calabar Teaching Hospital, Calabar, Nigeria

**Keywords:** ultra-portable, digital x-ray, computer-aided detection, tuberculosis, active case finding, Nigeria

## Abstract

**Background:**

The ultra-portable digital x-ray (UPDX) with computer-aided detection (CAD) is a new technology aimed at bringing tuberculosis (TB) screening innovations to hard-to-reach communities and strengthening the active case-finding (ACF) interventions for TB. As TB control measures remain critical globally and in Nigeria, the country acquired and rolled out 10 UPDX with computer-aided detection (CAD4TB) software in eight states. This study seeks to evaluate the efficiency and impact of the UPDX with CAD4TB for TB case finding in Nigeria.

**Methods:**

A retrospective cross-sectional study involving the review of records of individuals 4 years and above who had presented for TB screening during ACF activities conducted using the UPDX with CAD4TB between January 2022 and September 2022.

**Results:**

A total of 94,694 subjects aged 4 years and above were screened for TB with an average presumptive TB proportion of 10.84 ± 5.42% and 10% confirmed TB (*r* = 0.684, *p* = 0.03 and *r*(df) = 0.867, *p* = 0.001). The number needed to screen (NNS) to find one TB case in Northern Nigeria was 39 as against 37 for the South (*χ*^2^ = 108, *p* = 0.25), with correlations (*r* = −0.422, *p* = 0.17 and *r*(df) = −0.575, *p* = 0.05). Similarly, a comparison of the number needed to test (NNT) to find one TB case in Northern and Southern Nigeria gave a North total of four against a South total of five (*χ*^2^ = 60, *p* = 0.3), with correlations (*r* = −0.033, *p* = 0.92 and *r*(df) = −0.212, *p* = 0.51). Among the TB cases confirmed, 3.4% were asymptomatic with cough and fever absent in 18.2% and 83.2%, respectively. The average time to diagnosis (TTD) was 2.0 ± 1.04 days while the average time to treatment (TTT) was 4.2 ± 1.14 days, with 50.6% receiving same-day diagnosis and 34.5% receiving same-day treatment. The cumulative risks of radiation exposure on healthcare workers using UPDX with CAD4TB and adhering to personal protective practices were found to be low.

**Conclusion:**

We documented the usefulness of UPDX with CAD showing a high TB prevalence and test positivity rate, with significant burden of subclinical TB. This has highlighted the need to scale up its use for ACF for TB and select CAD thresholds for both children and adults.

## 1 Introduction

Tuberculosis (TB) is a preventable and treatable disease but it remains a deadly infectious disease. In 2022, the World Health Organization (WHO) estimated that 10.6 million people developed TB worldwide. Despite available cheap and effective treatment, TB still accounts for millions of active diseases and deaths worldwide (1). Nigeria is still ranked sixth globally and first in Africa in terms of TB burden (1). With over 200,000 missing TB cases, there is a need for targeted disease surveillance if this gap is to be closed. These missing TB cases are majorly accounted for by underdiagnosis due to both paucity and underutilization of screening, and diagnostic tools and equitable access opportunities to TB diagnostic platforms remain key to achieving TB control in Nigeria. Despite all these, TB case finding has remained passive, mostly limited to health facilities as symptomatic cases present for treatment. This has been grossly inadequate to meet TB elimination targets. The WHO has issued guidelines on systematic screening for active case finding (ACF), and an increasing number of countries are investing in national policies for ACF (2–4). Active case-finding (ACF) strategies are one of the key components of models designed to find the missing TB cases, especially in hard-to-reach underserved communities (4, 5). KNCV Tuberculosis Foundation Nigeria and Institute of Human Virology Nigeria (IHVN) through funding from USAID are implementing the Stop TB Partnership's Introducing New Tools Project (iNTP). This project procured 10 ultra-portable digital chest x-ray (UPDX) with computer-aided detection (CAD) for tuberculosis software to bring TB screening innovations to the doorsteps of hard-to-reach communities in Nigeria. This is in line with actions advocated by the WHO in the End TB Strategy to bridge the gap in TB case finding through the scale-up of the deployment of new tools for TB diagnosis and treatment (6).

The UPDX with CAD plays an important role in the screening and triage of pulmonary TB (PTB) and can guide the effective use of diagnostic testing to improve case detection and cost-efficiency. CAD products use artificial intelligence (AI) to analyze CXR images for the presence of abnormalities suggestive of PTB and can improve the feasibility and performance of CXR for TB screening and triage (7). For the iNTP project, the UPDX systems in use are the Delft Light (Delft Imaging, the Netherlands), coupled with CAD4TB software (version 7, Delft Imaging, the Netherlands). The software produces an abnormality score that can be used to signal probable TB and trigger further diagnostic evaluation. The score ranges between 0 and 100, and a threshold of 50 was set initially for the iNTP project to determine presumptive TB (8).

The UPDX with CAD provides an advantage of early detection of TB abnormalities, hence reducing the risk of community transmission. A large proportion of persons with active TB do not present with classical symptoms, and so the UPDX is used for ACF interventions in hard-to-reach communities to facilitate early diagnosis and treatment of TB. Tuberculosis screening activities using the UPDX with CAD are guided using a parallel screening algorithm where all clients undergo both symptom and CXR screening. The CAD threshold score was set at 50 at roll-out based on a review of available data at the time (8). Any client that screened positive for either the symptom or CXR CAD screening was classified as presumptive for TB. Further evaluation was conducted on the presumptive cases by collecting sputum samples for bacteriological tests using any of the molecular WHO-recommended rapid diagnostic tests (mWRDs). With the availability of CAD technology, the potential for rapidly scaling up UPDX for TB systematic screening and triaging has become a reality (9).

Current TB control measures, including early identification of TB disease, prompt, and appropriate treatment and case-holding of TB cases, continue to be critical in Nigeria and the world at large. This study seeks to evaluate the impact of the UPDX with CAD4TB intervention for ACF for TB in Nigeria by assessing the number needed to screen (NNS) and number needed to treat (NNT), to diagnose one person with active TB in Nigeria, comparing the efficiencies of UPDX with CAD4TB between Southern and Northern Nigeria, ascertaining the proportion of asymptomatic bacteriologically positive cases that were detected using the UPDX with CAD4TB, evaluating the results of the use of UPDX with CAD4TB in diagnosing TB in individuals 4 years and above, determining the average time to diagnosis (TTD) and time to treatment (TTT) of TB cases following screening using UPDX, and measuring the cumulative radiation exposure on healthcare workers.

The aim of the study is to evaluate the efficiency and impact of the ultra-portable digital x-ray with CAD4TB intervention for TB case finding in Nigeria.

The specific objectives are as follows:

1. To assess the efficiency of the ultra-portable digital x-ray with CAD4TB [number needed to screen (NNS) and number needed to treat (NNT), to diagnose one person with active TB for ACF in Nigeria]
2. To compare the efficiencies of ultra-portable digital x-ray with CAD4TB between Southern and Northern Nigeria
3. To ascertain the proportion of asymptomatic bacteriologically positive cases that were detected using the digital x-ray and CAD intervention
4. To evaluate the results of the use of ultra-portable digital x-ray with CAD4TB in diagnosing TB in individuals 4 years and above
5. To determine the average time to diagnosis and time to treatment of TB cases diagnosed following screening using Delft Light
6. To measure the cumulative radiation exposure on healthcare workers

## 2 Materials and methods

### 2.1 Study design

A retrospective cross-sectional study was conducted involving the review of records of individuals who had presented for TB screening or participated in community-based ACF activities using the UPDX with CAD between January 2022 and September 2022.

### 2.2 Study setting

**Figure 1:**
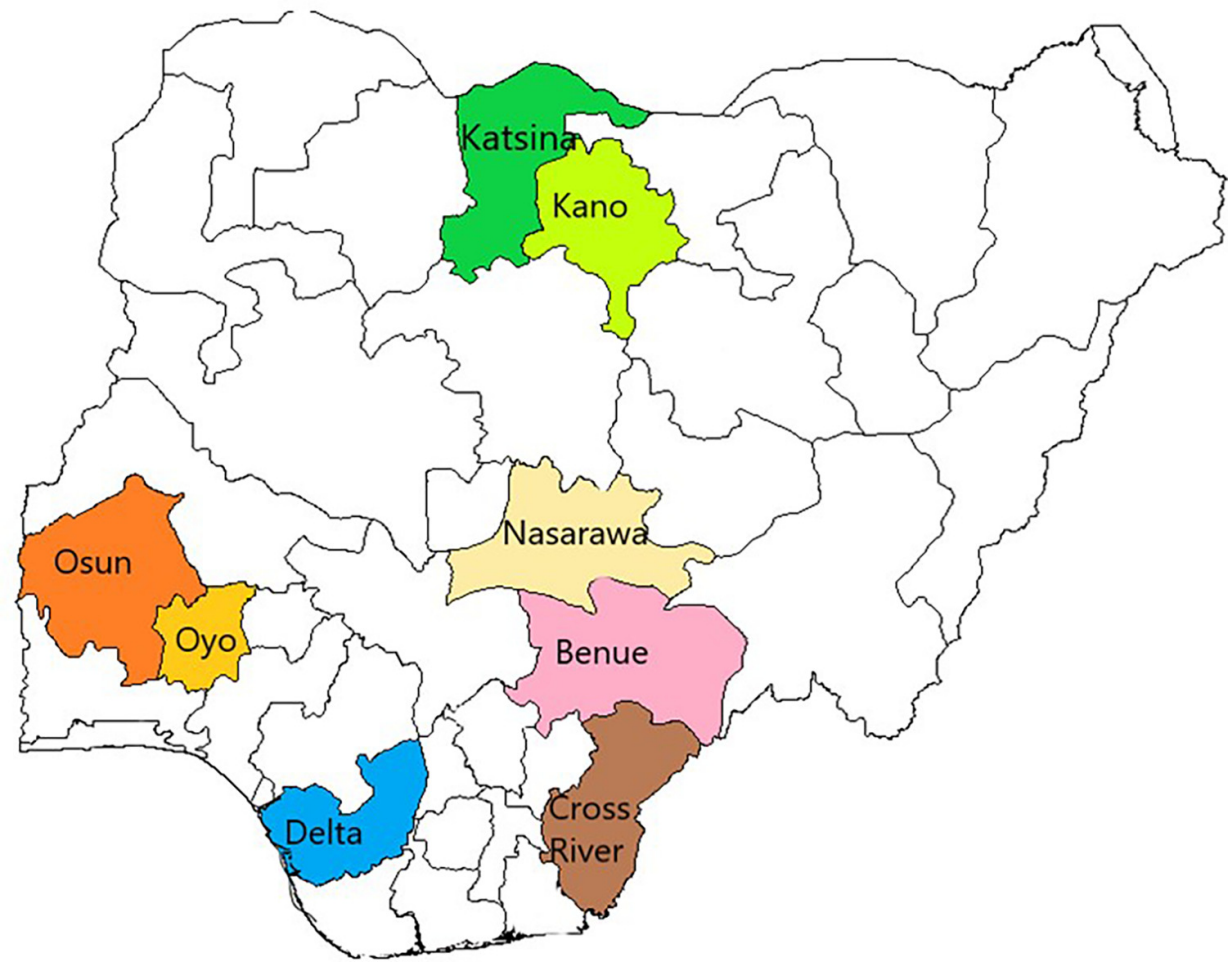
Map of Nigeria indicating states implementing the UPDX with CAD under the iNTP.

This study was conducted in eight purposively selected states where the UPDX machines were deployed for ACF for TB. They included four states (Katsina, Kano, Nasarawa, and Benue) in the Northern part of Nigeria with an average TB case notification rate of 94/100,000 population and four states (Delta, Cross Rivers, Osun, and Oyo States) in the southern part of Nigeria with an average TB case notification of 112/100,000 population (10, 11) (Figure 1).

Ten (10) UPDX machines were deployed across these eight states participating in the study, with Kano and Osun states having two machines each and other States having one each. These UPDX machines are used in suburban slums, rural communities, and other special population groups [e.g., prisons and internally displaced persons (IDP) camps] with each UPDX team comprising two staff (an associate and an assistant). Sputum and stool samples collected during the screening were transported to the nearest GeneXpert facilities for processing, and those with positive results were referred for treatment.

### 2.3 Study population

The target study population was children aged 4 years and above, adults living in suburban slums and rural communities, and other special population groups (e.g., prisons and IDP camps) in the eight implementing states. Radiographers using the UPDX to assess radiation dosage and safety were also included as part of the study population. All participants who were eligible and screened with the UPDX and CAD for TB and who were further evaluated bacteriologically or clinically with results were included in the study. Pregnant women or participants with medical conditions that are not compatible with performing a CXR were excluded.

Radiographers handling the UPDX used personal protective equipment (PPE) while operating the UPDX. They were also expected to follow radiation safety measures which include maintaining a specific distance from the UPDX generator while in use and wearing dosimeters which were measured regularly to assess the level of radiation exposure. This information was documented in the records for data extraction (further details are described in Section 2.6.3).

### 2.4 Sample size determination

There was no sample size calculation for this study as all the participants with clearly documented information during the study period were included.

### 2.5 Sampling approach

The purposive sampling technique used for this study implied that individuals with confirmed TB status according to bacteriological or radiological results (both TB and non-TB cases) were retrospectively identified and extracted from national TB registers and the data contained in the UPDX systems. No recruitment was needed as only a review of medical records was performed. Additionally, no consent was needed since it is a retrospective study but a waiver was obtained from the National Health Research Ethical Committee (NHREC) for the study.

### 2.6 Data collection

Excel-based data extraction forms/tools were developed by the research team to extract data of the eligible participants from the national TB recording and reporting tools and the data stored in the UPDX systems. This included participants' demographic and clinical data. Names and other unique patient identifiers were excluded during the data collection, with individuals anonymized by assigning each individual a unique patient ID.

#### 2.6.1 Study tools and variables

The quantitative structured data extraction tools/forms were used to extract sociodemographic information which included patient ID, age, sex, and screening location. The clinical variables extracted included symptoms (fever, cough, weight loss, night sweats), history of previous TB treatment, HIV status, CAD score, outcome of bacteriological evaluation (positive/negative), mode of diagnosis (bacteriological/clinical), and availability of the molecular WHO-recommended diagnostic tests (mWRD availability). Other variables collected included the number of clients screened, number of presumptive TB generated, number of presumptive evaluated, number of TB cases diagnosed (DS-TB and RR-TB), and number of TB cases placed on treatment (see Supplementary Data Sheet S1 for data collection tool/variables).

#### 2.6.2 Data management and statistical analysis plan

Data quality checks were done daily by cross-checking discrepancies and completeness of data on all variables. The research team conducted a real-time form review and validated missing/erroneous entries on-site by referring to the medical records and exported the data into SPSS for analysis.

Data were analyzed using Microsoft Excel and IBM SPSS software version 26. Summary statistics were generated and presented in frequency tables and charts. Means and standard deviations were used for quantitative continuous variables. Descriptive statistics were generated and presented in frequency tables and charts. The normally distributed continuous variables were described using mean and standard deviation or median and range, where appropriate. Inferential statistics were used to test for association between variables.

Bivariate analysis using the Pearson correlation coefficient was used to assess the factors associated with NNS, NNT, TTD, or TTT across the study sites. The NNS to diagnose one person with active TB is the inverse of the prevalence of detectable TB in the group, and in this case, it was the inverse of the prevalence of detectable TB (all bacteriologically diagnosed, clinically diagnosed, and RR-TB cases) among total clients screened with the UPDX. The NNT to diagnose one person with active TB is the inverse of test positivity which in this case is a product of the inverse of the detectable TB (all bacteriologically diagnosed, clinically diagnosed, and RR-TB cases) among total presumptive TB cases. Both univariate and multivariable logistic regression models were performed on the study outcomes, and ≤0.05 level of significance was used to select variables to include in the multivariate analysis model. Stepwise logistic regression modeling was undertaken to identify predictive variables for the proportion of asymptomatic bacteriologically positive cases detected using the UPDX and CAD intervention, average TTD and TTT of TB cases diagnosed following screening using UPDX, efficiency of the UPDX with CAD4TB ACF (NNS and NNT to diagnose one person with active TB), and geographical differences in the NNS and NNT between northern and southern UPDX machines. These were carried out by an automatic process while controlling for the confounding effect of other covariates, and all factors with *p* ≤ 0.05 were then considered significant.

#### 2.6.3 Description of equipment

The Delft Light Backpack is a UPDX system that can be deployed in various settings. It comprises an x-ray generator TR 90/20 (manufactured by Mikasa), an x-ray detector CXDI 702-C with accompanying application software, an HP laptop, and a tablet. Each component has its own built-in battery, allowing off-grid use for a limited period. The generator battery has a capacity and offers approximately 200 exposures. The duty cycle of the Delft Light is 1:60, meaning one exposure lasting approximately one-fifth of a second is possible every 12 s. Other key components include supporting stands, exchangeable battery/battery charger, solar panel, carrying case/bag, and radiation safety equipment. All Delft Light components (x-ray generator, detector, detector stand, console, and accessories, including CAD4TB box) can be packed into a single backpack, except the generator stand, which has its own bag for transport. In remote TB screening settings, all system components (x-ray generator, detector, workstation, and CAD4TB box) can be recharged from a portable Mobisun solar panel (with a built-in power bank). The unit, which is water resistant, takes 16 h to fully charge in direct sunlight. Alternatively, it can be charged from the grid in roughly 2.5 h.

The radiation safety equipment comprises one protective lead apron without a thyroid shield (2 mmPb thickness), five portable radiation hazard warning signs, and 10 shock detection stickers. The radiation risks to patients, workers, and the public from the medical use of x-rays conform to radiation safety standards. The UPDX manufacturers report it delivers a radiation dose well below 0.1 mSv, which corresponds to 1/30th of the average annual radiation dose from the environment (3 mSv) and 1/10th of the annual dose limit for the public which is 1 mSv (2). The dosimeter reading was conducted by the Nigeria Nuclear Regulatory Agency and compared against the annual exposure limit.

## 3 Results

### 3.1 The DLB screening cascade for the period January to September 2022

**Figure 2:**
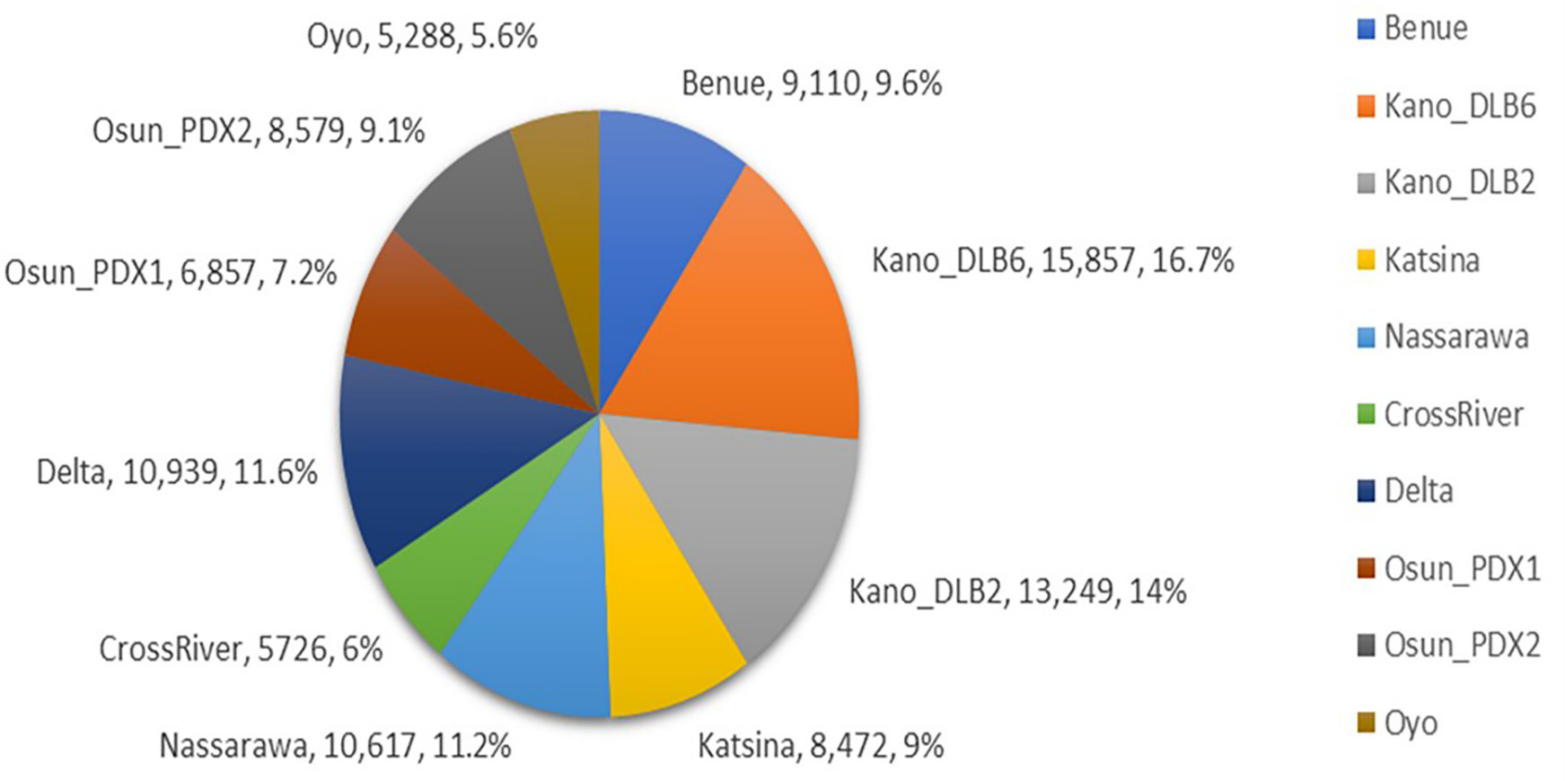
Performance of clients screened with CXR showing the proportions by location.

This study made use of retrospective program data from the implementation scale-up of 10 UPDX with CAD in the eight states to assess the efficiency and impact of its use on TB case finding in Nigeria. A total of 94,694 subjects were screened with CXR, among whom were 49,789 males with a male–female ratio of 1:1.1. Across the 10 UPDX sites, the number of clients screened with CXR by location ranged from 5,288 to 15,857 with an average of 9,469 ± 3,313. Kano_DLB6 screened the highest number of clients (15,857, 16.7%), followed by Kano_DLB2 (13,249, 14%), while the lowest performers were Osun_PDX1, Cross River, and Oyo with 6,857 (7.2%), 5,726 (6%), and 5,288 (5.6%) respectively (Figure 2).

**Figure 3:**
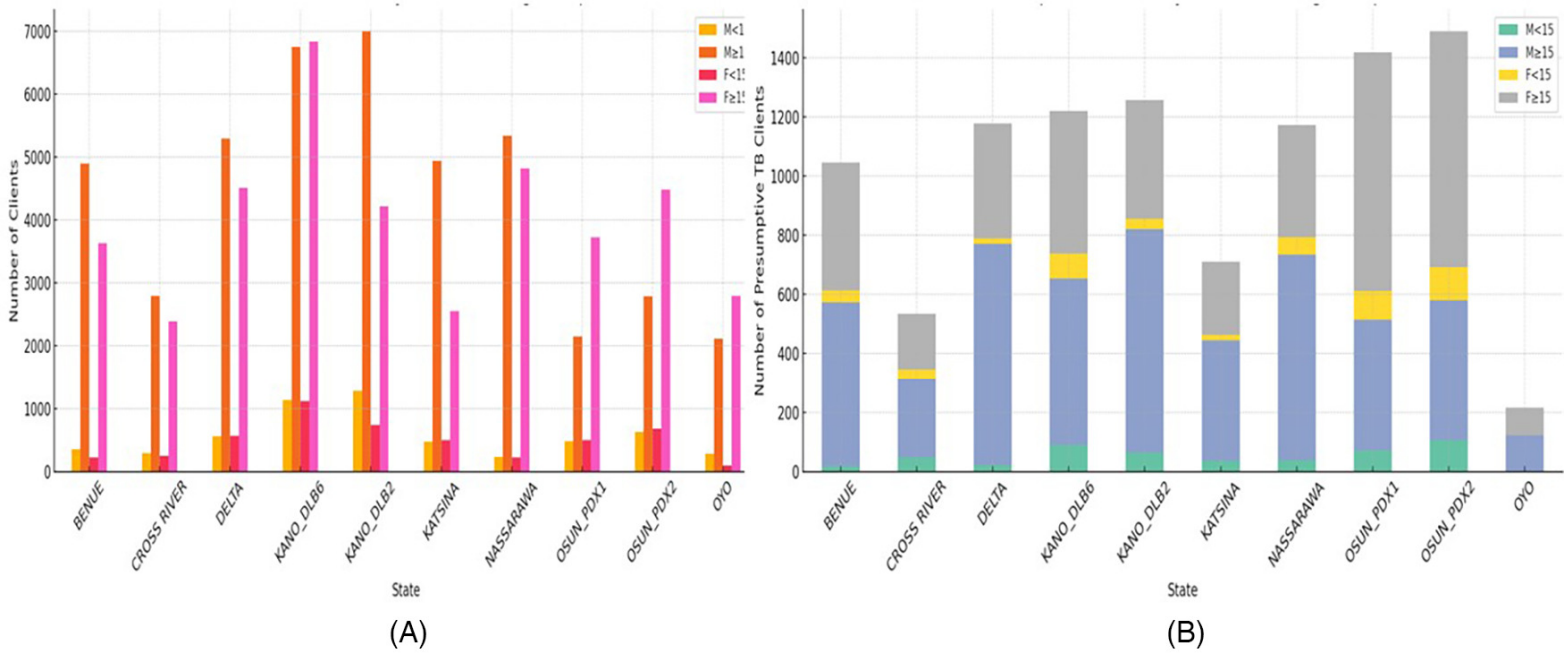
Total clients screened with UPDX and the resultant presumptive TB and their percentages by age groups and sex in all sites and regions from January to September 2022. **(A)** Total clients screened by state, sex, and age groups. **(B)** Presumptive TB clients by state, sex, and age groups.

An analysis of the presumptive TB cases arising from the clients’ CXR screening cascade showed a total of 10,236 presumptive TB cases with a range of 216–1,489 and a mean of 1,023.6 ± 408.5 per site, which was further disaggregated by age groups and sex as shown in Figure 3. A comparison of clients screened across all locations and presumptive TB did not reveal any positive correlation (*r* = 0.543, *p* = 0.105 and *r*(df) = 0.491, *p* = 0.15). The proportion of presumptive TB among the clients screened was 9.4% in the North and 12.9% in the South with a mean of 10.84 ± 5.4, which was further disaggregated by location, age group, and sex as shown in Figure 3.

**Figure 4:**
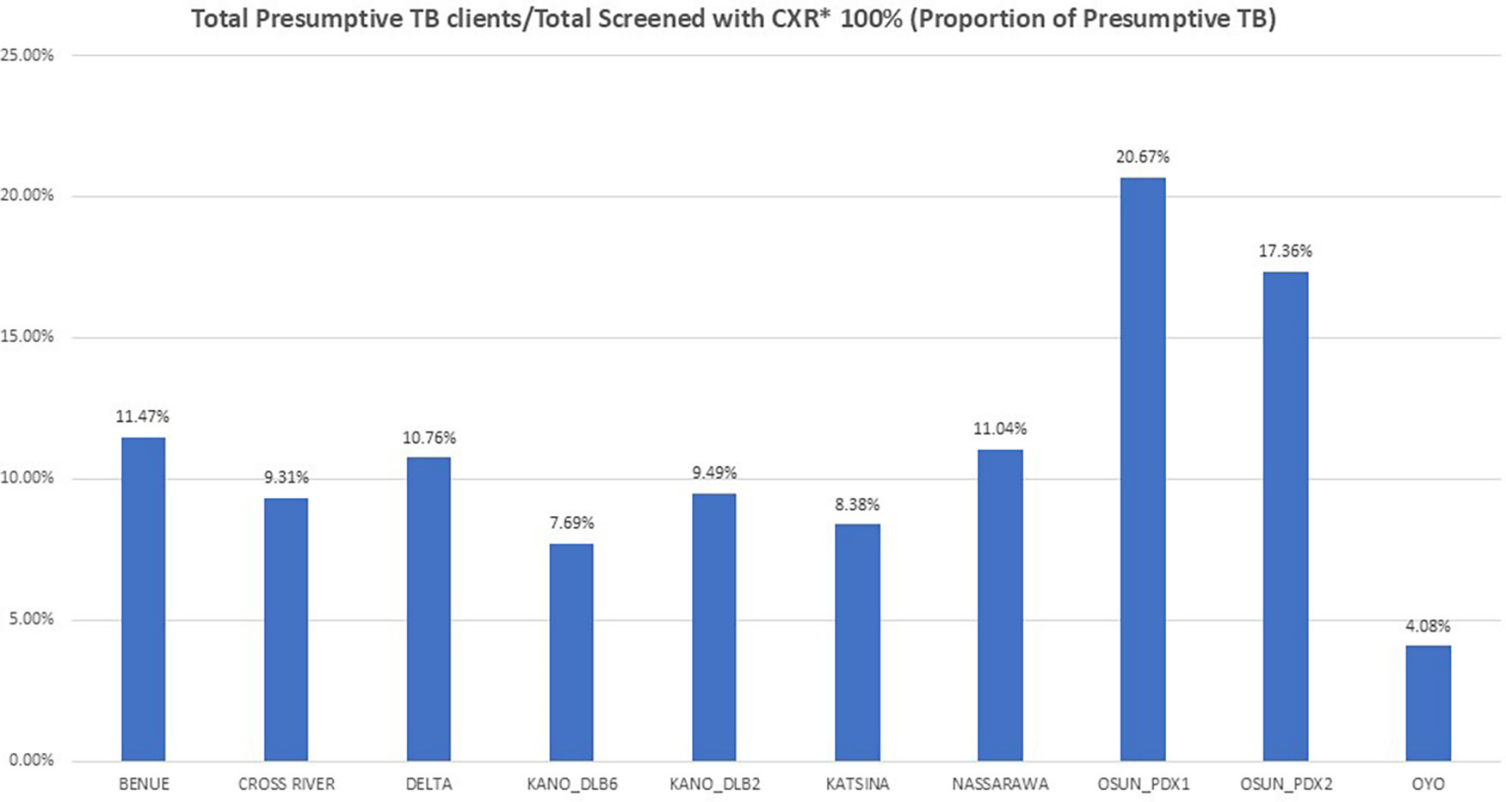
Proportion of presumptive TB by location from January to September 2022.

The proportion of presumptive TB over the study period across all locations showed Osun_PDX1 having the highest at 20.7% followed by Osun_PDX2 at 17.4% while Kano_DLB6 and Oyo had the lowest at 7.7% and 4.1%, respectively (Figure 4).

The screening then cascaded down to the total presumptive TB bacteriologically tested, total bacteriologically diagnosed, total clinically diagnosed, total rifampicin resistant, and total started on treatment as shown in Table 1. The presumptive TB cases bacteriologically tested ranged from 216 to 1,451 with a mean of 998 and an average testing rate of 97.5%. The bacteriologically diagnosed cases also ranged between sites from 19 to 265 (mean = 99), with a diagnosis rate of 10%. The highest mode of TB diagnosis was clinical in 1,386 (59.3%), followed by bacteriologically positive DS-TB (DS-TB) cases in 920 (39.4%), while bacteriologically positive RR-TB (RR-TB) was present in 31 (1.3%). The results of the Pearson correlation analysis revealed a positive correlation between the presumptive TB bacteriologically tested and bacteriologically diagnosed patients (*r* = 0.684, *p* = 0.03), and the Spearman correlation was also positive (*r*(df) = 0.867, *p* = 0.001). The yield of rifampicin resistance (RR) ranged from 0% to 1% with a national average of 0.3% (Table 1).

**Table 1:** TB screening cascade for ultra-portable digital x-ray machines with CAD.

UPDX machine	Total CXR CAD screened total (%)	Total presumptive TB clients (%)	Total presumptive TB bact. tested (%)	Total bact. diagnosed patients (%)	Total clinically diagnosed patients (%)	Total RR-TB (%)	Total started on treatment (%)
Benue	9,110 (9.6)	1,045 (11.47)	1,045 (100))	46 (4.4)	283 (27.1)	2 (0.2)	328 (99.1)
Cross River	5,726 (6)	533 (9.31)	471 (88.4)	19 (3.6)	52 (11)	0 (0.0)	62 (87.3)
Delta	10,939 (11)	1,177 (10.76)	1,071 (91)	47 (4)	174 (16.3)	0 (0.0)	209 (94.6)
Kano_DLB6	15,857 (16.7)	1,220 (7.69)	1,208 (99}	72 (5.9)	273 (22.6)	4 (0.33)	347 (99.4)
Kano_DLB2	13,249 (14)	1,257 (9.49)	1,244 (99)	116 (9.2)	248 (20)	0 (0.0)	361 (99.2)
Katsina	8,472 (9)	710 (8.38)	710 (100)	89 (12.5)	148 (20.8)	1 (0.14)	236 (99.2)
Nassarawa	10,617 (11)	1,172 (11.04)	1,172 (100)	66 (5.6)	110 (9.4)	1 (0.09)	177 (100)
Osun_PDX1	6,857 (7)	1,417 (20.67)	1,394 (98.4)	231 (16.3)	44 (3.2)	5 (0.36)	276 (98.6)
Osun_PDX2	8,579 (9.1)	1,489 (17.36)	1,451 (97.5)	265 (17.8)	72 (5)	15 (1.0)	337 (95.7)
Oyo	5,288 (5.6)	216 (4.08)	216 (100)	35 (16.2)	52 (24.1)	1 (0.5)	81 (92)
Total	94,694	10,236 (10.8)	9,982 (97.5)	986 (10)	1,466 (14.7)	29 (0.3)	2,414 (97.3)

The number needed to screen (NNS) to diagnose one person with active TB is the inverse of the prevalence of detectable TB in the group, and in this case, it was the inverse of the prevalence of detectable TB (all bacteriologically diagnosed, clinically diagnosed, and RR-TB cases) among total clients screened with the UPDX (Table 2). The average NNS for the study was 38, ranging from 24 in Osun and 28 in Benue to the highest of 60 in Nasarawa and 81 in Cross River states. A comparison of NNS in Northern and Southern Nigeria gave an NNS of 39 for the North as against 37 for the South (*χ*^2^ = 108, *p* = 0.25), with correlations (*r* = −0.422, *p* = 0.17 and *r*(df) = −0.575, *p* = 0.05). There was no significant difference in the numbers needed to screen between sites in the North and South. On the other hand, the number needed to test (NNT) to diagnose one person with active TB is the inverse of test positivity which in this case is a product of the inverse of the detectable TB (all bacteriologically diagnosed, clinically diagnosed, and RR-TB cases) among total presumptive TB cases (Table 2). The average NNT for the study was 4, with lower NNT ranging from 2 in Oyo to 3 in Benue, Kano, and Katsina States, compared with the highest NNT of 6 in Nasarawa and 7 in Cross River States. A comparison of NNT in Northern and Southern Nigeria gave a North total of 4 against a South total of 5 (*χ*^2^ = 60, *p* = 0.3), with correlations (*r* = −0.033, *p* = 0.92, and *r*(df) = −0.212, *p* = 0.51, respectively) (Table 2).

**Table 2:** The number needed to screen (NNS) and number needed to test (NNT) to diagnose one person with active TB.

States/UPDX	Number needed to screen (NNS) to diagnose one person with active TB			Number needed to test (NNT) to diagnose one person with active TB		
	Total clients screened with x-ray and CAD	Total TB cases diagnosed (DS-TB, CLIN, RR-TB)	NNS	Total presumptive TB clients	Total TB cases diagnosed (DS-TB, CLIN, RR-TB)	NNT
North_Benue	9,110	331	28	1,045	331	3
North_Kano_DLB6	15,857	349	45	1,220	349	3
North_Kano_DLB2	13,249	364	36	1,257	364	3
North_Katsina	8,472	238	36	710	238	3
North_Nassarawa	10,617	177	60	1,172	177	6
Subtotal northern region	57,305 (60.5)	1,459	39	5,404	1,459	4
Pearson *R* (approximate significance)			**−0.422 (*p* = 0.17)**			**−0.033 (*p* = 0.92)**
South_Cross River	5,726	71	81	533	71	7
South_Delta	10,939	221	49	1,177	221	5
South_Osun_PDX1	6,857	280	24	1,417	280	5
South_Osun_PDX2	8,579	352	24	1,489	352	4
South_Oyo	5,288	98	54	216	98	2
Subtotal southern region	37,389 (39.5)	1,022	37	4,832	1,022	5
Total	94,694	2,481	**38**	10,236	2,481	**4**

* Statistically significant or *P* < 0.05.

### 3.2 Attributes of patients diagnosed with TB within the study period (January to September 2022)

**Figure 5:**
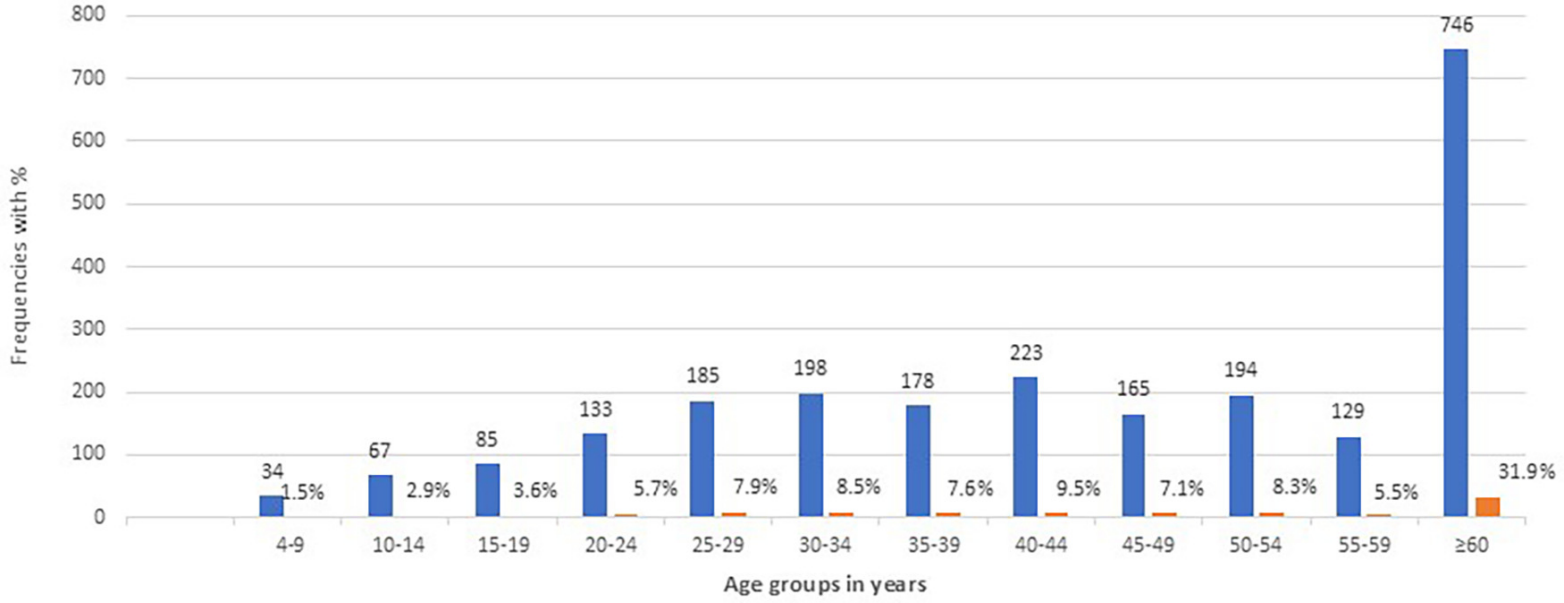
Age groups of diagnosed patients and their frequency distributions from January to September 2022.

There were 2,337 patients diagnosed with TB, of whom 1,493 (63.9%) were males. The mean age was 46.7 ± 19.8 years with a median of 45 years. The age groups of the patients and their frequencies included children aged 4–14 years (101, 4.4%), adults aged 15–59 years (1,490, 63.8%), and the elderly aged ≥60 years (746, 31.9%). The frequency distribution of their 5-year age groups showed a gradual increase of TB cases from early childhood and adolescence through several active adult age groups, peaking at 40–44 years of age before gradually decreasing to just before 60 years of age, but not reaching child and adolescent levels. The burden then escalated in the elderly aged 60 years and above (Figure 5).

The distribution of TB cases among the 2,337 patients across the 10 sites was also documented, with the highest cases coming from Osun_PDX2 (338, 14.5%) and Benue (334, 14.3%), followed closely by Kano_DLB2 (302, 12.9%), Osun_PDX1 (275, 11.8%), Kano_DLB6 (271, 11.6%), and Katsina (252, 10.8%) and then Delta (219, 9.4%), Nasarawa (177, 7.6%), Oyo (97, 4.2%), and Cross River recording (72, 3.1%). The burden of TB in the northern states was 1,336 (57.2%), while that in the Southern states was 1,001 (42.8%).

The screening location with the highest number of TB cases was the community with 1,883 patients (80.6%), followed by prisons with 306 (13.1%), refugee/IDP camps with 64 (2.7%), health facilities including both primary and secondary with 34 (1.5%), schools with 23 (1%), and others with 27 (1.2%). A comparison of screen location and age groups showed children aged 4–14 years constituting 82 (4.4%) of the community cases, 6 (2%) of the prison cases, 13 (56.5%) of the cases in schools, and 0% in each of refugee/IDP camps, facilities, and others (*χ*^2^ = 390.54, *p* = 0.000) which was significant. The Pearson correlation analysis did not reveal a positive correlation between screen location and age groups (*r* = −0.019, *p* = 0.35), but the Spearman correlation was negative between the two variables (*r*(df) = −0.091, *p* = 0.000). Additionally, the association between screen location and sex showed males constituted 61.7% of the community cases, 89.2% in the prisons, 34.4% in the refugee/IDP camps, 47.8% in schools, 32.4% in facilities, and 55.6% in others (*χ*^2^ = 131.27, *p* = 0.000) which was significant. The results of the Pearson correlation analysis did not reveal a positive correlation between screen location and sex (*r* = 0.03, *p* = 0.199), but the Spearman correlation was negative between the two variables (*r*(df) = −0.07, *p* = 0.000).

Analysis of the availability of the molecular WHO-recommended diagnostic tests (mWRD availability) among the diagnosed patients revealed only one TB case (0%) clinically diagnosed where no mWRD was available, 943 (40.4%) diagnosed where the mWRD was available in the same community as ACF, 789 (33.8%) diagnosed where the mWRD was available in a different town/community in the local government area (LGA), and 604 (25.8%) diagnosed where mWRD was available coupled with Truenat or TB LAMP for ACF. An association between age groups and mWRD availability showed none of the children aged 4–14 years were diagnosed where there was no mWRD availability, 37 (3.9%) were diagnosed where the mWRD was available in the same community as ACF, 47 (6%) diagnosed where the mWRD was available in a different town/community in LGA, and 17 (2.8%) were diagnosed where mWRD was available coupled with Truenat or TB LAMP for ACF (*χ*^2^ = 85.54, *p* = 0.000). Moreover, mWRD availability was correlated with sex, and no male was diagnosed where there was no availability, 633 (67.1%) of the males were diagnosed where the mWRD was available in the same community as ACF, 419 (53.1%) were diagnosed where the mWRD was available in a different town/community in LGA, and 441 (73%) of them were diagnosed where mWRD was available coupled with Truenat or TB LAMP for ACF (*χ*^2^ = 67.09, *p* = 0.000).

Concerning the mode of confirmation of TB diagnosis, the highest was clinical in 1,386 (59.3%), followed by bacteriologically positive DS-TB (DS-TB) cases in 920 (39.4%), while bacteriologically positive RR-TB (RR-TB) was present in 31 (1.3%). An association between TB diagnosis and age showed that 54 (5.9%) were DS-TB, 3 (9.7%) were RR-TB, and 44 (3.2%) were clinically diagnosed TB (*χ*^2^ = 43.6, *p* = 0.004) which was significant. The Pearson correlation analysis revealed a positive correlation between TB diagnosis and age groups (*r* = −0.079, *p* = 0.000), but the Spearman correlation was mildly negative between the two variables (*r*(df) = −0.069, *p* = 0.001). A comparison of TB diagnosis and sex showed male predominancies of 541 (58.8%) DS-TB, 16 (51.6%) RR-TB, and 936 (67.5%) clinically diagnosed TB (*χ*^2^ = 20.31, *p* = 0.000) which was significant. The Pearson correlation analysis revealed a weak negative correlation between the mode of confirmation of TB diagnosis and age groups (*r* = −0.089, *p* = 0.000), and the Spearman correlation was weakly negative between the two variables (*r*(df) = −0.090, *p* = 0.000). The correlation between TB diagnosis and screen location showed DS-TB diagnosis of 748 (81.3%) in the community, 101 (11%) in prisons, 5 (0.5%) in refugee/IDP camps, 20 (2.2%) in schools, 29 (3.2%) in facilities, and 17 (1.8%) in others (*χ*^2^ = 125.9, *p* = 0.000). The Pearson correlation analysis revealed a weak negative correlation between the mode of confirmation of TB diagnosis and age groups (*r* = −0.071, *p* = 0.001).

Moreover, when TB diagnosis was correlated with mWRD availability, it showed 1 (0.1%) diagnosed DS-TB with no availability of mWRD, 314 (34.1%) diagnosed where the mWRD was available in the same community as ACF, 468 (50.9%) diagnosed where the mWRD was available in a different town/community in LGA, and 137 (14.9%) diagnosed where mWRD was available coupled with Truenat or TB LAMP for ACF. In addition, RR-TB was not detected where there was no availability of mWRD, 3 (9.7%) were diagnosed where the mWRD was available in the same community as ACF, 21 (67.8%) were diagnosed where the mWRD was available in a different town/community in LGA, and 7 (22.6%) were diagnosed where mWRD was available coupled with Truenat or TB LAMP for ACF (*χ*^2^ = 247.95, *p* = 0.000, and *r* = −0.044, *p* = 0.03).

When CAD scores were categorized into <50 (negative) and ≥50 (positive) among those diagnosed with TB, those who scored ≥50 constituted 1,614 (69.1%) while those who scored <50 constituted 723 (30.9%). A comparison of CAD score and TB diagnosis showed 482 (52.4%) of DS-TB were CAD positive while 438 (47.6%) were CAD negative. Among the clinically diagnosed TB cases, 1,122 (81.0%) were CAD positive, and 264 (19.0%) negative, but among the RR-TB cases, only 10 (32.3%) were CAD positive, and the remaining 21 (67.7%) were CAD negative (*χ*^2^ = 2,780.63, *p* = 0.000). Analysis of age group vs. CAD4TB score for diagnosed TB patients showed 65 (9.7%) children aged <15 years had CAD scores <50 and 34 (2.1%) had scores ≥50 while among those children aged ≥15 years, 605 (90.3%) had CAD scores <50 and 1,596 (97.9%) had scores ≥50 (*χ*^2^ = 96.168, *p* = 0.000). Further disaggregation among the children showed that 18 (27.7%) of children aged 4–9 years diagnosed with TB had CAD scores <50 while 14 (41.2%) of them had scores ≥50. Among children aged 10–14 years diagnosed with TB, 47 (72.3%) had scores <50 while 20 (58.8%) had scores ≥50.

The asymptomatic bacteriologically positive cases detected were 80 (3.4%), and none of the bacteriologically positive cases had all four symptoms (cough, night sweat, weight loss, and fever) simultaneously present. Analysis of the frequency and proportion of different symptoms revealed that cough was absent in 427 (18.2%), night sweats in 1,164 (49.8%), weight loss in 1,140 (39.1%), and fever in 1,945 (83.2%) of the patients diagnosed with TB (Table 3).

**Table 3:** Symptoms among patients diagnosed with TB.

Symptoms	Frequency	Percentage
Asymptomatic		
Yes	80	3.4
No	2,257	96.7
Cough		
Absent	427	18.2
Present	1,910	81.8
Night sweat		
Absent	1,164	49.8
Present	546	23.4
Weight loss		
Absent	1,140	39.1
Present	573	24.6
Fever		
Absent	1,945	83.2
Present	386	16.5

The results of using UPDX with CAD4TB in diagnosing TB in individuals aged 4 years and above were also evaluated in relation to TB-related symptoms and their various associations. Table 4 shows that the types of TB-related symptoms differed and were significantly related to the screening location among patients. Cough was significantly most common among those who were screened in the refugee/IDP camps, followed by prisons, communities, and facilities (*p* = 0.000). Night sweat was significantly absent among those screened in refugee/IDP camps, prisons, and communities (*p* = 0.000). Moreover, those screened in refugee/IDP camps and communities had significantly absent weight loss (*p* = 0.000). Fever was consistently absent across the board, but this was not significant (*p* = 0.100) as shown in Table 4.

**Table 4:** Association between screening location and type of symptom among patients.

Symptoms	Screen location						Chi-square	*p*-value
	Community	Facility	Prison	Refugee or IDP	School	Others		
	*N* = 1,883	*n* = 34	*n* = 306	*N* = 64	*N* = 23	*N* = 27		
Cough								
Absent	360 (19.1)	7 (20.6)	45 (14.7)	0 (0.0)	8 (34.8)	7 (25.9)	50.91	0.000*
Present	1,523 (80.9)	27 (79.4)	261 (85.3)	64 (100)	15 (65.2)	20 (74.1)		
Night sweat								
Absent	940 (49.9)	9 (26.5)	167 (54.6)	44 (68.8)	0 (0.0)	4 (14.8)	239.92	0.000*
Present	407 (21.6)	0 (0.0)	117 (38.2)	20 (31.3)	0 (0.0)	2 (7.4)		
Unknown	536 (28.5)	25 (73.5)	22 (7.2)	0 (0.0)	23 (100)	21 (77.8)		
Weight loss								
Absent	948 (50.3)	8 (23.5)	132 (43.1)	50 (78.1)	0 (0.0)	2 (7.4)	302.88	0.000*
Present	402 (21.3)	1 (2.9)	152 (49.7)	14 (21.9)	0 (0.0)	4 (14.8)		
Unknown	532 (28.3)	25 (73.5)	22 (7.2)	0 (0.0)	23 (100)	21 (77.8)		
Fever								
Absent	1,551 (82.4)	34 (100)	262 (85.6)	51 (79.7)	23 (100)	24 (88.9)	15.97	0.100
Present	326 (17.3)	0 (0.0)	44 (14.4)	13 (20.3)	0 (0.0)	3 (11.1)		
Unknown	6 (0.3)	0 (0.0)	0 (0.0)	0 (0.0)	0 (0.0)	0 (0.0)		

* Statistically significant.

When considering the relationship between symptoms and bacteriological status, a significantly lower proportion of those with DS-TB were found among patients with an absence of night sweat (*p* = 0.000), weight loss (*p* = 0.000), and fever (*p* = 0.000). On the other hand, patients without cough accounted for the highest proportion of those with DS-TB (Table 5).

**Table 5:** Relationship between symptoms and bacteriological status.

Symptoms	Bacteriologically positive				Chi-square test	*p*-value
	Yes DS-TB	Yes RR-TB	No	Total		
	*n* = 920	*N* = 31	*n* = 1,386	*N* = 2,337		
Fever						
Absent	831 (42.7)	25 (1.3)	1,089 (56)	1,945 (100.0)	66.26	0.000*
Present	89 (23.1)	5 (1.3)	292 (75.6)	386 (100.0)		
Unknown	0 (0.0)	1 (16.7)	5 (83.3)	6 (100.0)		
Night sweats						
Absent	284 (24.4)	4 (0.3)	876 (75.3)	1,164 (100.0)	614.62	0.000*
Present	141 (25.8)	7 (1.3)	398 (72.9)	546 (100.0)		
Unknown	495 (78.9)	20 (3.2)	112 (17.9)	627 (100.0)		
Weight loss						
Absent	260 (22.8)	6 (0.5)	874 (76.7)	1,140 (100.0)	607.94	0.000*
Present	168 (29.3)	6 (1.0)	399 (69.6)	573 (100.0)		
Unknown	492 (78.8)	19 (3.0)	113 (18.1)	624 (100.0)		
Cough						
Absent	250 (58.5)	8 (1.9)	169 (39.6)	427 (100.0)	87.26	0.000*
Cough <2 weeks	301 (33.2)	13 (1.4)	592 (65.3)	906 (100.0)		
Cough >2 weeks	369 (36.8)	10 (1.0)	625 (62.3)	1,004 (100.0)		

* Statistically significant.

Among the CAD-positive patients, a significant proportion recorded no weight loss, while 87.8% of them had weight loss (*p* = 0.000). Moreover, 69.3% of patients had no fever as against 68.4% who developed fever (*p* = 0.000) as shown in Table 6.

**Table 6:** Relationship between symptoms and CAD score.

Symptoms	CAD score			Chi-square test	*p*-value
	≥50	<50	Total		
	*n* = 1,614	*n* = 723	*N* = 2,337		
Cough					
Absent	209 (48.9)	218 (51.1)	427 (100.0)	1,650.0	0.93
Cough <2 weeks	650 (71.7)	256 (28.3)	906 (100.0)		
Cough >2 weeks	755 (75.2)	249 (24.8)	1,004 (100.0)		
Fever					
Absent	1,347 (69.3)	598 (30.7)	1,945 (100.0)	66.26	0.000*
Present	264 (68.4)	122 (31.6)	386 (100.0)		
Unknown	3 (50.0)	3 (50.0)	6 (100.0)		
Night sweats					
Absent	745 (64.0)	419 (36.0)	1,164 (100.0)	1,810.0	0.112
Present	470 (86.0)	76 (14.0)	546 (100.0)		
Unknown	207 (33.0)	420 (67.0)	627 (100.0)		
Weight loss					
Absent	946 (83.0)	194 (17.0)	1,140 (100.0)	4,101.29	0.000*
Present	503 (87.8)	70 (12.2)	573 (100.0)		
Unknown	274 (43.9)	350 (56.1)	624 (100.0)		

* Statistically significant.

In Table 7, patients with no history of night sweats (OR = 0.155; 95% CI: 0.111–0.218) compared with those with an unknown history of night sweat were significantly less likely to have bacteriological positive results. Patients with no weight loss in comparison with those with weight loss (OR = 0.604; 95% CI: 0.458–0.795) and with an unknown history of weight loss (OR = 0.447; 95% CI: 0.306–0.652) were significantly less likely to have bacterially positive results. However, fever and cough were not independent predictors of a bacteriological positive result (Table 7).

**Table 7:** Binary logistic regression of symptoms as predictors of bacteriological status among patients.

Variable	Reference category	Odds ratio	95% confidence interval		*p*-value
			Lower	Upper	
Fever: present	No fever	0.954	0.744	1.223	0.712
Fever: unknown	No fever	0.776	0.556	1.085	0.138
Night sweats: present	No night sweats	1.014	0.778	1.321	0.919
Night sweats: unknown	No night sweats	0.155	0.111	0.218	0.000*
Weight loss: present	No weight loss	0.604	0.458	0.795	0.000*
Weight loss: unknown	No weight loss	0.447	0.306	0.652	0.000*
Cough: <2 weeks	No cough	1.126	0.837	1.515	0.431
Cough: >2 weeks	No cough	1.150	0.864	1.532	0.338

* Statistically significant.

The average time to diagnosis (TTD) was 2.0 ± 1.04 days while the average time to treatment (TTT) was 4.2 ± 1.14 days. Following the screening, 1,183 (50.6%) of the patients had zero day (same day) of diagnosis, 524 (22.4%) were diagnosed at 1–2 days, 477 (20.4%) were diagnosed at 2–7 days, and 6.5% were diagnosed after 7 days. For the TTT, 808 (34.8%) of the patients received their anti-TB treatment on Day 0 (same day) of being screened, 514 (22.1%) between 1 and 2 days, 594 (25.6%) from 2 to 7 days, and 405 (17.4%) after 7 days (*χ*^2^ = 613.52, *p* = 0.000). Sixteen (0.7%) did not start treatment. The Pearson correlation analysis revealed a positive correlation between TTD and TTT (*r* = 0.223, *p* = 0.000), and the Spearman correlation was also positive between the two variables *r*(df) = 0.212, *p* = 0.000 (Table 8).

**Table 8:** Time to diagnosis and time to treatment.

Time in days	Time to diagnosis (TTD) frequency (%)	Time to treatment (TTT) frequency (%)	Chi-square	*p*-value
Day zero (<1 day)	1,183 (50.6%)	808 (34.8%)	613.52	0.000*
1–2 days	524 (22.4%)	514 (22.1%)		
Above 2–7 days	477 (20.4%)	594 (25.6%)		
Above 7 days	153 (6.5%)	405 (17.4%)		
No treatment		16 (0.7%)		

* Statistically significant.

**Figure 6:**
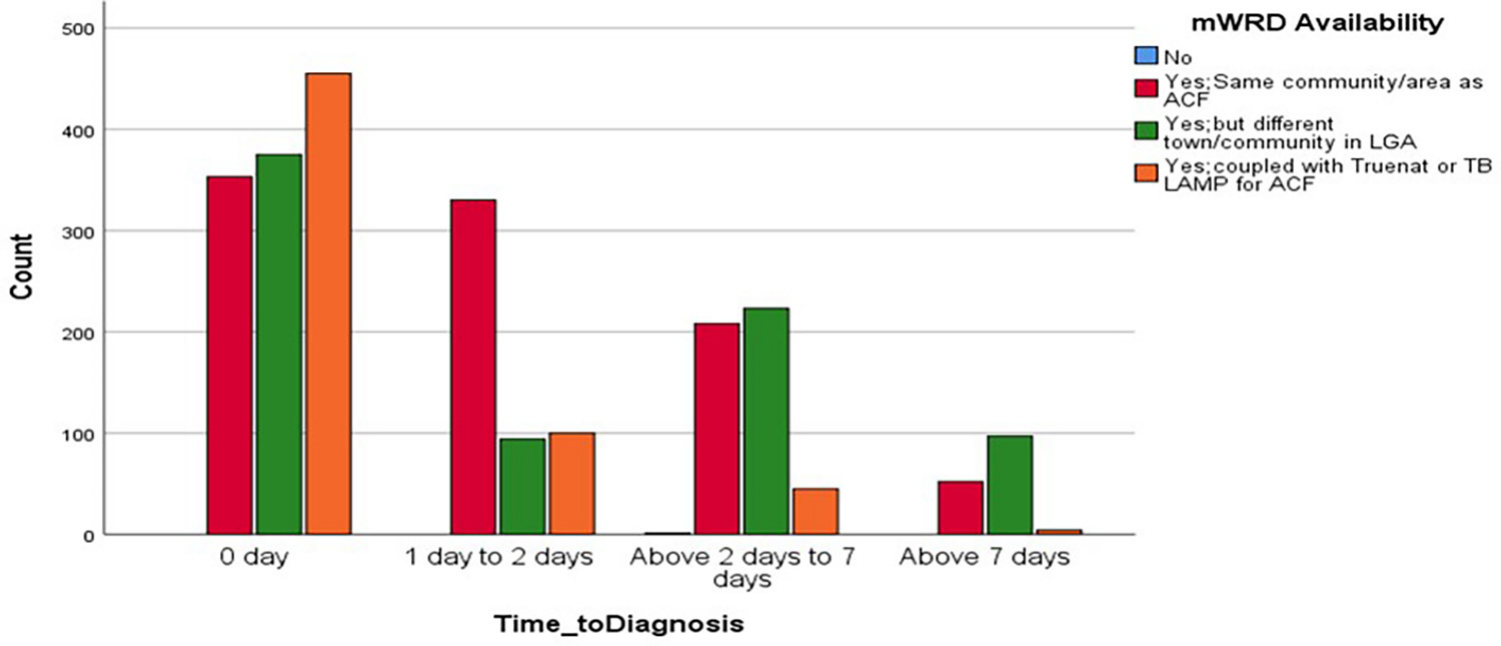
Association between TTD and MWRD availability.

When TTD and mWRD were compared, a significant proportion of patients got same-day results irrespective of the mRWD availability, 353 (29.8%) were diagnosed where the mWRD was available in the same community as ACF, 375 (31.7%) were diagnosed where the mWRD was available in a different town/community in LGA, and 455 (38.5%) were diagnosed where mWRD was available coupled with Truenat or TB LAMP for ACF. At 1–2 days, the proportion of diagnosed cases increased sharply to 330 (63%) for those whose mWRD availability was in the same community as ACF (Figure 6).

**Figure 7:**
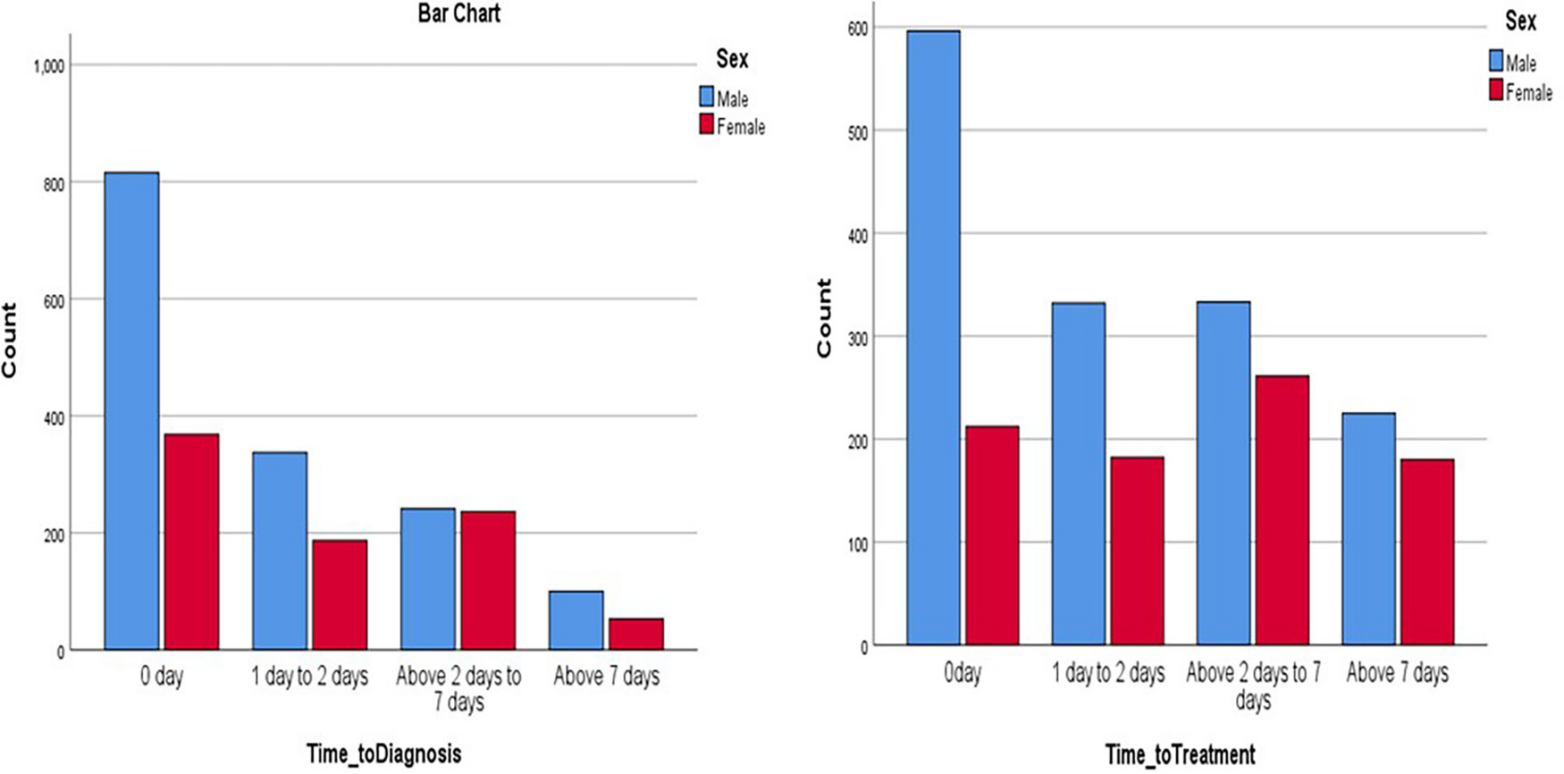
Association between sex of patients and TTD and TTT.

When TTD and TTT were correlated with sex, a significantly greater proportion of males were generally diagnosed and treated across all the time strata compared with the females (Figure 7).

### 3.3 Radiation measurement and exposure on healthcare workers

An evaluation of the measurement and the cumulative radiation exposure on healthcare workers assessed the risks of radiation exposure in various aspects. A lead apron was worn during screening by all (100%) 10 radiographers. The thickness of the apron worn ranged from 0.25 to 0.35 mmPb with a mean of 0.32 ± 0.048. The distance of the radiographer from the generator ranged from 1.5 m (60%) to 6 m in 10% of cases, and a remote trigger was used with the Delft Light UPDX to take exposures from this distance. The dosimeter badge was always worn by all the radiographers, and the most common mode of wearing it/location worn was outside apron (top part) in 50% of cases followed by inside apron (top part) in 40% of cases. None wore the badge on the inside apron (mid-section). All (100%) the radiographers measured the dosimeter quarterly, and the radiation dosimetry report from the last radiation exposure for that quarter ranged from <0.01 to 0.37 mSv with a mean of 0.19 ± 0.025 mSv, while the number of working hours per screening day ranged from 4 to 8 h with an average of 5.70 ± 1.42 h.

The cumulative risks of radiation exposure were further assessed by associating distance from the generator in meters and the location of the badge worn, and this was not significant (*χ*^2^ = 6.92, *p* = 0.546). A relationship between the apron thickness (mmPb) and the location of the badge worn was also not significant (*χ*^2^ = 1.429, *p* = 0.490). A comparison of the distances from the generator across all the UPDX machines was also not significant (*χ*^2^ = 40, *p* = 0.3). All 10 UPDX machines were compared with the location of the badge worn (*χ*^2^ = 20.00, *p* = 0.33), radiation exposure according to dosimeter readings (*χ*^2^ = 70.00, *p* = 0.254), working hours (*χ*^2^ = 44.00, *p* = 0.31), and apron thickness in mmPb (*χ*^2^ = 10.00, *p* = 0.35), and these were not significant. Relating the working hours and apron thickness (*χ*^2^ = 4.44, *p* = 0.36), distance from the generator (*χ*^2^ = 14.17, *p* = 0.31), location of the badge worn (*χ*^2^ = 5.75, *p* = 0.67), and radiation exposure (*χ*^2^ = 21.67, *p* = 0.92) were all not significant. An assessment of the risk of exposure due to the duration of working hours vs. several variables including apron thickness in mmPb, distance in meters, badge wear frequency, location of badge worn, radiation report, apron worn, badge measured, badge worn, and frequency of measure and their frequencies and cumulative exposures were not found to be significant.

## 4 Discussion

The UPDX with CAD4TB is a new technology aimed at bringing diagnostic innovations to the doorstep of hard-to-reach communities and strengthening the ACF for TB in the country. Our study focused on evaluating the impact of this intervention on screening, early diagnosis, and treatment of patients diagnosed with TB under program conditions. The proportion of presumptive TB among clients screened ranged from 4.1% to 20.7% across the 10 sites with a mean of 10.8%. A similar intervention conducted in Nigeria documented the usefulness of the UPDX in ACF and showed a presumptive TB yield of 8.6% (12). Other studies have shown such interventions resulting in improved case detection and the provision of access to high-quality people-centered TB care owing to better portability of the UPDX (13, 14). The treatment rate in our study was 97.3%, similar to studies in the region documenting 98.2% (15) and increased treatment rate from 14% to 56% (16) following such interventions. This strategy using UPDX with CAD4TB, combined with TB diagnosis particularly through portable molecular diagnostics using Truenat and TB LAMP, has been shown to increase TB case detection and treatment rates in this and other studies (12, 15–18) and has highlighted great potential of optimizing current TB case-finding policies (18).

The UPDX sites with the highest numbers screened did not necessarily have the highest presumptive TB yield. Although Kano_DLB6, Kano_DLB2, Nasarawa, and Delta recorded the highest numbers of clients screened, Osun_PDX1 had the highest presumptive TB yield of 20.7% followed by Osun_PDX2 with 17.4%, while Kano_DLB6 and Oyo had the lowest with 7.7% and 4.1%, respectively. Therefore, beyond the numbers screened, purposive or targeted ACF in selected high-risk/TB-endemic populations is very important and should be adopted by the program as documented in other studies (12, 17, 19).

The efficiency of the UPDX with CAD4TB intervention in Nigeria was also evidenced by the NNS and NNT in diagnosing one person with active TB. The average NNS and NNT across all 10 UPDX machines were 38 and 4, respectively. This is significantly better than the NNS and NNT from interventions that apply only symptom screening during ACF activities (12). The NNS among the different states ranged from 24 in Osun to 81 in Cross River, while the NNT ranged from 3 in Benue, Kano, and Katsina to 7 in Cross River. Regional differences in the efficiencies of the UPDX with CAD4TB intervention in Nigeria have been documented by other studies (12, 15), but none compared the geographical differences between the northern and southern parts of the country. This study showed there was no significant difference in the average NNS and NNT between states in the northern and southern parts of Nigeria. The efficiency could be further improved by calibrating the CAD threshold since those with CAD scores <50 constituted 30.9% of diagnosed TB cases, and this could result in missed cases if CAD was used at this threshold for triage alone. This has implications for threshold score selection and could be helpful for other implementers with similar results (20, 21). Moreover, among the DS-TB cases, 52.4% were CAD positive while 47.6% were CAD negative. There is a need for better optimization of the CAD threshold to avoid undertreatment and missing cases (22).

There is very limited evidence base regarding CAD use in children with most studies on CAD conducted on populations aged 15 years and above (14, 23–25). Analysis from screening children <15 years in our study showed only 2.1% of those diagnosed with TB had CAD scores ≥50. This is very similar to a study conducted in the Niger Delta region of Nigeria where 2.1% of children aged below 15 years with confirmed TB had CAD scores ≥60 (12). Moreover, the CAD scores in our study were negative (<50) in 9.7% of children aged below 15 years with bacteriologically confirmed TB, indicating that the appropriate threshold may likely be below 50. A further disaggregation among the children showed 27.7% of children aged 4–9 years diagnosed with TB had CAD scores <50 while 41.2% of them had scores ≥50. Among children 10–14 years diagnosed with TB, 72.3% had scores <50 while 58.8% of them had scores ≥50. This raises concerns about the sensitivity of the CAD threshold level of 50 in use which was also reported in other studies (25). Because of diagnostic differences in the various subpopulations, there is a need to select CAD threshold instead of using predetermined scores. Unfortunately, CAD has not yet been validated for TB screening or triage in children partly due to the paucity of data and resources to do so (26).

Our study seems to be one of the first few to report data on CAD use in children aged 4–14 years in Nigeria, as current WHO recommendations excluded children aged <15 years from the use of CAD systems despite having several CAD products in the market, including CAD4TB used in this study (27). Since childhood TB diagnosis is known to be challenged by both paucibacillary TB and a wide spectrum of clinical disease (28, 29), the use of lateral CXR to access hilar opacities in addition to clinical information may improve the diagnostic accuracy, but the current CAD products read only anteroposterior CXR views (26). Although there is a paucity of data on the performance of CAD scores in children aged <15 years, it is now no secret that there is a need to better document the algorithm performance among different subpopulations including children (24, 30).

The UPDX with CAD4TB intervention in our study showed bimodal age disaggregated frequencies and distribution of TB at age 40–44 years and a tall peak in the elderly aged above 60 years. Other studies on TB case-finding interventions have shown similar bimodal distribution of TB at age 20–24 and 70–74 years (31) and men having two peaks at age 45–54 and >65 years (32). Bimodal age risk groups of TB have been observed to facilitate targeted, age-specific policies, and actions for healthcare and disease management. The WHO has since recommended age disaggregated data from birth to the oldest reached age, but this has not been adhered to as observed by some studies (33, 34). This lack of standardized age disaggregation adversely affects the interpretation of results of health interventions and the comparison of data across indicators in countries and regions (34).

Not all clients screened were bacteriologically TB tested as shown in our study with an average testing rate of 97.5%, similar to a study done in the same region testing 96.5% of those screened (15). Although CXR is a useful tool in diagnosing TB, it should not stand alone to establish a diagnosis without an attempt at bacteriological confirmation of TB (2, 12, 13, 15, 17, 35). The bacteriological TB diagnosis rate in our study was 10%, while clinically diagnosed was 14.7%. Other studies documented a higher bacteriological diagnosis rate of 15.3% with 4.9% (24), 22.9% (36), and between 36.1% and 79.7% of RR-TB (18). The high rate of clinical diagnosis in this study buttresses the fact that the UPDX with CAD4TB is so useful/sensitive that in early infection, it goes ahead of the organisms to pick up the initial pathological changes that are visible on the CXR but not bacteriologically confirmed (18).

The proportion of asymptomatic bacteriologically positive cases that were detected using the UPDX with CAD4TB was 3.4%, and these would have been missed if screening utilized the WHO four symptom screen alone, although another study documented 18.2% (37). Cough, which is the strength of the TB symptom complex, was absent in 18.2% of our patients, as against other studies where cough was absent in 10.3% (24) and approximately 50% of bacteriologically confirmed TB across populations in Asia and Africa (18). Other studies have documented TB without weight loss (38) and TB with absent weight loss in 37.2% (24), similar to our study where weight loss was absent in 39.1%. Studies have documented TB without fever in 20.5% (24), coupled with the absence of fever in 83.2% and night sweats in 49.8% in our study, further raising concerns about the TB burden being missed by the current WHO symptom screening (18). Studies that screened for TB irrespective of symptoms had a 3.7% higher yield than strategies focusing on symptomatic patients (17). Moreover, another study showed variations in the strengths of associations between these symptoms and TB diagnosis, except cough >2 weeks which was significant (3).

Subclinical TB cases are gaining increasing attention due to their high potential to cause TB transmission (37), and TB experts and stakeholders need to recognize and appreciate the implications of subclinical TB and offer strategies to address it. Other studies have documented that groups at high risk of subclinical TB are epidemiologically similar to those with active TB, including those residents in TB-endemic areas and those with previous TB (37). The issue at hand now is the extent to which clients at risk for subclinical TB should be prioritized for testing and treatment and the cost-effectiveness of such interventions. The capacity of the UPDX with CAD to facilitate the detection of subclinical TB demonstrates its utility in early detection and breaking the transmission of TB disease.

The average TTD of TB cases using UPDX with CAD4TB in our study was 2.0 ± 1.04 days and TTT was 4.2 ± 1.14 days. This is similar to a previous facility-based study in the same country where TTD ranged from 1.9 to 2.9 days and TTT ranged from 1.1 to 3.9 days (16). A scale-up of this will go a long way to end TB. There was a preponderance of males generally diagnosed and treated across all the time strata compared with the females, as documented in other studies where males are generally overrepresented in TB notification (15). This demonstrates that men should be considered a key population for TB as they have a higher risk, and the UPDX with CAD could be an important tool for finding and treating more men with TB.

Sixteen (0.7%) of the patients had not started treatment during the study period. They either refused outrightly, denied diagnosis, or traveled out of the community and could not be traced prior to the study. Contrary to the belief that attrition is next to zero in community-based studies, it does occur and should be kept in view with a high index of suspicion.

Our study also aimed to measure the cumulative radiation exposure on UPDX operators and assessed the risks of radiation exposure in various aspects. All 10 UPDX machines were assessed by the location of badge worn, radiation reports, working hours, apron thickness in mmPb, relating the working hours and apron thickness, distance from the generator, location of the badge worn and radiation report, and all parameters and correlations were found not to be significant. Radiation exposure depends on the imaging equipment used, its exposure settings, thickness of the client's body, and the PPE (37, 39). A lead apron was worn during screening by all users in our study which agrees with another study reporting that HCWs showed good adherence to radiation safety measurements (40). However, another study decries a low attitude and low formal education in radiation safety among HCWs using radiation equipment (41). The system operated below the manufacturer's reported emissions and leakage parameters, below the threshold doses for participants and health workers, and below international guidelines for radiographers (14). Even when the risk of exposure during working hours was associated with several variables, occupational and radiation exposure dose limits were well below limits for international safety guidelines indicating a high radiation safety profile for the UPDX equipment (39). As a reference standard, the decision to expose any client to radiation, no matter how minimal, must adhere to the principles of justification and optimization. Justification means the procedure should do more good than harm and optimization means the doses should be kept as low as possible and that the benefits of the x-rays outweigh any potential risks from radiation exposure (2).

### 4.1 Limitations of the study

There were some gaps noted in the documentation and reporting of some symptoms and the HIV status of screened clients. This is a known disadvantage of retrospective data and the gaps in documentation created the significant unknown categories. These unknown categories may have affected the study results and is advisable that the UPDX with CAD outings should strengthen the collaboration with the HIV testing team, among others for accurate assessment of HIV positivity.

### 4.2 Recommendations/outlook for future implementers

1. Considering the usefulness of the UPDX with CAD intervention and especially the CXR positivity rate in these patients with bacteriologically positive TB, there is a need to scale up the use of UPDX with CAD4TB for active case finding for TB in both children and adults in more states.
2. The burden of subclinical TB is grossly underestimated as prevalence surveys generally do not capture individuals who are symptom-negative, x-ray–negative, bacteriologically confirmed TB. There is a need for increased awareness and a high index of suspicion of the existence of subclinical tuberculosis for prioritization of TB care and appropriate resource allocation to populations at risk.
3. The UPDX with CAD outings should strengthen the collaboration with the HIV testing team, among others, for accurate assessment of HIV positivity. Also, an increase in UPDX team members for improved pretest counseling and extraction of detailed history will be more useful to reduce these high “unknown” rates.
4. The radiation safety concerns of using UPDX with CAD4TB are well noted and found to be overestimated from the results of this study where the measurements and risks of radiation exposures are not significant and well below recommended levels. There's therefore a need to update the guidelines on international radiation safety standards for manufacturers, TB policymakers, and program managers using UPDX with CAD and any such innovative new technologies.

### 4.3 Actionable guidelines for implementing UPDX in other regions/similar settings

1. Scaling up the use of UPDX with CAD4TB for active case finding for TB in both children and adults
2. Promoting active case finding with parallel screening algorithm for both children and adults to identify subclinical TB
3. Strengthening the collaboration with the HIV screening team among others
4. Increasing the UPDX team members for improved pretest counseling and extraction of detailed history to reduce these high “unknown” rates
5. Updating the guidelines on international radiation safety standards for manufacturers, TB policymakers, and program managers using UPDX with CAD and any such innovative new technologies.

## 5 Conclusion

Our study which focused on evaluating the impact of the UPDX with CAD4TB intervention on screening, early diagnosis, and treatment of patients diagnosed with TB under program conditions, documented the usefulness of UPDX with CAD intervention with a high prevalence and test positivity rate, thereby highlighting a need to scale up the use of UPDX with CAD4TB for active TB case finding in both children and adults in more states and regions. This study also highlighted the need for CAD threshold selection for adults and children, instead of using predetermined scores, for better optimization of the CAD threshold to avoid undertreatment and missing cases. Another highlight was the burden of subclinical TB which is usually grossly underestimated as prevalence surveys generally do not capture individuals who are symptom-negative, x-ray–negative, bacteriologically confirmed TB. Increased awareness and a high index of suspicion of the existence of subclinical tuberculosis are needed for the prioritization of TB care and appropriate resource allocation to populations at risk.

## Data Availability

The original contributions presented in the study are included in the article/Supplementary Material, further inquiries can be directed to the corresponding author.

## Supplementary material

Supplementary Material S1

DLB Screening cascade, diagnosed patients tools.

Supplementary Material S2

Radiation measurement tool.

## References

[1] World Health Organization. Global Tuberculosis Report 2023. Geneva: WHO/HTM/TB (2023); Licence: CC BY-NC-SA 3.0 IGO.

[2] World Health Organization. *Chest radiography in tuberculosis detection: summary of current WHO recommendations and guidance on programmatic approaches*. Geneva: World Health Organization (2016). Available at: https://iris.who.int/handle/10665/252424 (Accessed June 18, 2025).

[3] Morishita F Garfin AMCG Lew W Oh KH Yadav R-P Reston JC Bringing state-of-the-art diagnostics to vulnerable populations: the use of a mobile screening unit in active case finding for tuberculosis in Palawan, the Philippines. PLoS One. (2017) 12(2):e0171310. 10.1371/journal.pone.0171310 28152082 PMC5289556

[4] Saini V Garg K. Case finding strategies under National Tuberculosis Elimination Programme (NTEP). Indian J Tuberc (2020) 67:S101–6. 10.1016/j.ijtb.2020.09.029 33308653 PMC7526527

[5] Uplekar M Creswell J Ottmani SE Weil D Sahu S Lönnroth K. Programmatic approaches to screening for active tuberculosis. Int J Tuberc Lung Dis. (2013) 17(10):1248–56. 10.5588/ijtld.13.0199 24025375

[6] World Health Organization. The End TB Strategy. Geneva, Switzerland: World Health Organization (2015).

[7] World Health Organization. Determining the Local Calibration of Computer-assisted detection (CAD) Thresholds and Other Parameters: A Toolkit to Support the Effective Use of CAD for TB Screening. WHO TDR Toolkit. Geneva: World Health Organization (2021).

[8] Qin ZZ Barret R Ahmed S Sarker MS Paul K Adel ASS Comparing different versions of computer-aided detection products when reading chest x-rays for tuberculosis. PLoS Digital Health. (2022) 1(6):e0000067. 10.1371/journal.pdig.0000067 36812562 PMC9931298

[9] Khan FA Pande T Tessema B Song R Benedetti A Pai M Computer-aided reading of tuberculosis chest radiography: moving the research agenda forward to inform policy. Eur Respir J. (2017) 50(1):1700953. 10.1183/13993003.00953-2017 28705949

[10] National Population Commission, Abuja. Nigeria population estimate (2020). Available at: http://nationalpopulation.gov.ng/statistics/

[11] FMoH. National TB Leprosy and Buruli ulcer control programme, Federal Ministry of Health, Abuja. 2020 Annual TB Report (2021). Available at: https://ntblcp.org.ng resources 2020-annual-tb-report (Accessed June 18, 2025).

[12] Odume B Chukwu E Fawole T Nwokoye N Ogbudebe C Chukwuogo O Portable digital x-ray for TB pre-diagnosis screening in rural communities in Nigeria. Public Health Action. (2022) 12(2):85–9. 10.5588/pha.21.0079 35734009 PMC9176193

[13] Ogbuji QC Ojo OM Owoyomi O Anozie I Osho AJ Ladipo O Evaluation of house-to-house active tuberculosis case finding contribution to TB case notification in 10 states in Nigeria. Int J Med Sci Health Res. (2021) 5(4):18–29. 10.51505/ijmshr.2021.531

[14] Vo LNQ Codlin A Ngo TD Dao TP Dong TTT Mo HTL Early evaluation of an ultra-portable x-ray system for tuberculosis active case finding. Trop Med Infect Dis. (2021) 6:163. 10.3390/tropicalmed6030163 34564547 PMC8482270

[15] Eyo AS Obot VO Onyedinachi O Aguilera Vasquez N Bigio J Sanaie A A multi-faceted approach to tuberculosis active case finding among remote riverine communities in southern Nigeria. Int J Environ Res Public Health. (2021) 18:9424. 10.3390/ijerph18189424 34574349 PMC8472435

[16] Sani U Mustapha G Jumoke O Rupert E Nkemdilim C Emperor U FAST strategy - a sustainable administrative TB infection control measure in Nigeria: reducing time to TB diagnosis and enrolment to treatment. Sci J Public Health. (2016) 4(4):352–8. 10.11648/j.sjph.20160404.23

[17] Deya RW Masese LN Jaoko W Muhwa JC Mbugua L Horne DJ Yield and coverage of active case finding interventions for tuberculosis control: a systematic review and meta-analysis. Tuberc Res Treat. (2022) 2022(11):e9947068. 10.1155/2022/9947068 PMC9274229 35837369

[18] Frascella B Richards AS Sossen B Emery JC Odone A Law I Subclinical tuberculosis disease-a review and analysis of prevalence surveys to inform definitions, burden, associations and screening methodology. Clin Infect Dis. (2021) 73(3):e830–41. 10.1093/cid/ciaa1402 32936877 PMC8326537

[19] Ewa AU Cobham A Onwuteaka C Usoroh E Offor B. Targeted active case finding for tuberculosis in children in an urban community in Nigeria-preliminary findings. Abstract of proceedings. Annual General Meeting and Scientific Conference of the Nigerian Thoracic Society, Calabar, Nigeria: 21-22 November 2019. NJCD. (2020) 2(1):104.

[20] Qin ZZ Van der Walt M Moyo S Ismail F Maribe P Denkinger CM Computer-aided detection of tuberculosis from chest radiographs in a tuberculosis prevalence survey in South Africa: external validation and modelled impacts of commercially available artificial intelligence software. Lancet Digit Health. (2024) 6:e605–13. 10.1016/S2589-7500(24)00118-3 39033067 PMC11339183

[21] Vanobberghen F Keter AK Jacobs BKM Glass TR Lynen L Law I Computer-aided detection thresholds for digital chest x-ray interpretation in tuberculosis diagnostic algorithms. ERJ Open Res. (2023) 10(1):00508-2023. 10.1183/23120541.00508-2023 PMC10772898 38196890

[22] Palmer M Seddon JA van der Zalm MM Hesseling AC Goussard P Schaaf HS Optimising computer aided detection to identify intra-thoracic tuberculosis on chest x-ray in South African children. PLoS Glob Public Health. (2023) 3(5):e0001799. 10.1371/journal.pgph.0001799 37192175 PMC10187911

[23] John S Abdulkarim S Usman S Rahman MT Creswell J. Comparing tuberculosis symptom screening to chest x-ray with artificial intelligence in an active case finding campaign in northeast Nigeria. BMC Glob Public Health. (2023) 1:17. 10.1186/s44263-023-00017-2 39681894 PMC11622919

[24] Qin ZZ Ahmed S Sarker MS Paul K Adel ASS Naheyan T Tuberculosis detection from chest x-rays for triaging in a high tuberculosis-burden setting: an evaluation of five artificial intelligence algorithms. Lancet Digit Health. (2021) 3:e543–54. 10.1016/S2589-7500(21)00116-3 34446265

[25] Kagujje M Kerkhoff AD Nteeni M Dunn I Mateyo K Muyoyeta M. The performance of computer-aided detection digital chest x-ray reading technologies for triage of active tuberculosis among persons with a history of previous tuberculosis. Clin Infect Dis. (2023) 76:e894–901. 10.1093/cid/ciac679 36004409 PMC9907528

[26] Geric C Qin ZZ Denkinger CM Kik SV Marais B Anjos A The rise of artificial intelligence reading of chest x-rays for enhanced TB diagnosis and elimination. Int J Tuberc Lung Dis. (2023) 27(5):367–72. 10.5588/ijtld.22.0687 37143227 PMC10171486

[27] World Health Organization. WHO Consolidated Guidelines on Tuberculosis: Module 2: Screening: Systematic Screening for Tuberculosis Disease. Geneva: WHO (2021). 33822560

[28] National Tuberculosis, Leprosy and Buruli Ulcer Control Programme, Department of Public Health, FMoH. National tuberculosis, leprosy and Buruli ulcer management and control guidelines 7th edition (2021). p. 1–327.

[29] World Health Organization. WHO Consolidated Guidelines on Tuberculosis. Module 3: Diagnosis - Rapid Diagnostics for Tuberculosis Detection, 2021 Update. Geneva: World Health Organization (2021).

[30] Rahman MT Malik AA Amanullah F Creswell J. Improving tuberculosis case detection in children: summary of innovations and findings from 18 countries. J Pediatric Infect Dis Soc. (2022) 11(S3):S117–24. 10.1093/jpids/piac09 36103996

[31] Dong Z Wang Q-Q Yu S-C Huang F Liu J-J Yao H-Y Age–period–cohort analysis of pulmonary tuberculosis reported incidence, China, 2006–2020. Infect Dis Poverty. (2022) 11:85. 10.1186/s40249-022-01009-4 35902982 PMC9331155

[32] Daniel O Adejumo O Bamidele J Alabi A Gbadebo A Oritogun K. Social determinants of tuberculosis in Nigeria: an ecological approach. J Public Health Afr. (2022) 13(4):2215. 10.4081/jphia.2022.2215

[33] Aduh U Ewa A Sam-Agudu N Urhioke O Kusimo O Ugwu C Addressing gaps in adolescent tuberculosis programming and policy in Nigeria from a public health perspective. Int J Adolesc Med Health. (2021) 33(3):41–51. 10.1515/ijamh-2020-0293 33913304

[34] Diaz T Strong KL Cao B Guthold R Moran AC Moller A-B A call for standardised age-disaggregated health data. Lancet Healthy Longev. (2021) 2(7):e436–43. 10.1016/S2666-7568(21)00115-X. Erratum at *Lancet Healthy Longev*. (2021) 2(8):e458. doi: 10.1016/S2666-7568(21)00171-9 34240065 PMC8245325

[35] Ewa AU Essiet DF Monu SJU. Tuberculosis in children living amongst adults with tuberculosis at the tuberculosis and leprosy referral hospital, Eku, Nigeria. J Tuberc Res. (2015) 3:80–9. 10.4236/jtr.2015.33013

[36] Ochang EA Emanghe UE Ewa A Otu A Offor JB Odo M Evaluation of pulmonary tuberculosis case detection improvement with the deployment of XpertMTB/Rif in the tuberculosis control program of Cross River State, Nigeria. Int J Mycobacteriol. (2017) 6(1):94–6. 10.4103/2212-5531.201890 28317812

[37] Tang P Liang E Zhang X Feng Y Song H Xu J Prevalence and risk factors of subclinical tuberculosis in a low-incidence setting in China. Front Microbiol. (2022) 12:731532. 10.3389/fmicb.2021.73153 35087480 PMC8787132

[38] Ewa A Umana A Okoi N Akpan F Enyuma F. Tuberculosis in well-nourished children in Calabar, Nigeria. J Tuberc Res. (2015) 3(2):59. 10.4236/jtr.2015.32009

[39] Garcia-Sanchez A-J Angosto EAG Riquelme PAM Berna AS Ramos-Amores D. Ionizing radiation measurement solution in a hospital environment. Sensors. (2018) 18:510. 10.3390/s18020510 29419769 PMC5855489

[40] Abuzaid M Elshami W Hasan H. Knowledge and adherence to radiation protection among healthcare workers at operation theater. Asian J Sci Res. (2019) 12:54–9. 10.3923/ajsr.2019.54.59

[41] Alkhayal AM Alothman AS Alathel AH AlMaslamani A Alfehaid ON Alhassan IA Knowledge and attitude of radiation safety and the use of protective measures among healthcare workers in a tertiary center. Eur Rev Med Pharmacol Sci. (2023) 27:2047–51. 10.26355/eurrev_202303_31575 36930506

