# Supplementary material for: Impact of the use of the ultra-portable digital x-ray with CAD4TB for active case finding for tuberculosis in Nigeria
Source: Front Digit Health. 2025 Jun 30;7:1559203. doi: 10.3389/fdgth.2025.1559203 (PMC12257310; doi:10.3389/fdgth.2025.1559203)
Supplement: Supplementary file 1 [file Datasheet1.zip › Annex 2_Radiation Measurement tool.pdf]

Supplementary Material 2: Radiation measurement tool

Variable	Description	Data type	Codes/ranges
Apron_worn	Lead apron worn during screening (All the time; sometimes)	Numeric	0 = No<br>1 = Yes, Always<br>2 = Yes, Sometimes
Apron_Thickness	Thickness of apron	Numeric	Value
Distance	Distance of radiographer from Generator	Numeric	Value
Badge_worn	Dosimeter badge worn	Numeric	0 = No<br>1 = Yes
Badge_wear_frequency	Frequency of use of dosimeter badge	Numeric	0 = Always<br>1 = Forget sometimes (Specify)
Location_of_badge_worn	Mode of wearing dosimeter badge/ location worn	Numeric	0 = Outside apron (top part)<br>1 = Outside apron (Mid-section)<br>2 = Inside apron (Top part)<br>3 = Inside apron (Mid-section)
Badge_measured	Dosimeter measurement done?	Numeric	0 = No<br>1 = Yes
Frequency_of_measure	# of times measured within the last 6 months	Numeric	Value
Radiation_report	Last radiation exposure report	Numeric	Value
Working Hours	Average number of working hours per screening day	Numeric	Value
