# Supplementary material for: Impact of the use of the ultra-portable digital x-ray with CAD4TB for active case finding for tuberculosis in Nigeria
Source: Front Digit Health. 2025 Jun 30;7:1559203. doi: 10.3389/fdgth.2025.1559203 (PMC12257310; doi:10.3389/fdgth.2025.1559203)
Supplement: Supplementary file 1 [file Datasheet1.zip › Annex 1_DLB Screening Cascade and Diagnosed Patients tools.pdf]

Supplementary Material 1: Data collection instruments

DLB Screening Cascade Template

MONTH	Jan-22				Feb-22				Mar-22			
STATE	Male		Female		Male		Female		Male		Female	
	<15	15>	<15	15>	<15	15>	<15	15>	<15	15>	<15	15>
Clients screened with CXR												
Presumptive TB clients												
Presumptive tested bacteriologically test												
Bacteriologically diagnosed patients												
Clinically diagnosed patients												
RR TB												
Started on treatment												

MONTH	Apr-22				May-22				Jun-22			
STATE	Male		Female		Male		Female		Male		Female	
	<15	15>	<15	15>	<15	15>	<15	15>	<15	15>	<15	15>
Clients screened with CXR												
Presumptive TB clients												
Presumptive tested bacteriologically test												
Bacteriologically diagnosed patients												
Clinically diagnosed patients												
RR TB												
Started on treatment												

MONTH	Jul-22				Aug-22				Sep-22			
STATE	Male		Female		Male		Female		Male		Female	
	<15	15>	<15	15>	<16	15>	<16	15>	<16	15>	<16	15>
Clients screened with CXR												
Presumptive TB clients												
Presumptive tested bacteriologically test												
Bacteriologically diagnosed patients												
Clinically diagnosed patients												
RR TB												
Started on treatment												

DIAGNOSED PATIENTS DATA EXTRACTION TOOL

**Variable name**	**Description**	**Data type**	**Codes/ranges**
DLB/PDX Code	Name and State Code of DLB	String	Nil
Patient ID	Unique number used to identify participants	Numeric	1 >
CAD score	Numerical output score produced by a CAD reading	Numeric	0 – 100
Age/Age_gp	Classification of patient aged >4 years and over based on age groups	Numeric	4 – 100<br>4-9=0<br>10-14=1<br>15-19=2<br>20-24=3<br>25-29=4<br>30-34=5<br>35-39=6<br>40-44=7<br>45-49=8<br>50-54=9<br>55-59=10<br>≥60=11
Sex	Sex	Numeric	0= M
			1 = F
Screen Date	Date Patient was screened using the PDX_CAD	Numeric	dd/mm/yyyy

Screen Location	Location patient was screened	Numeric	0 = Community
			1 = Prison
			2 = Refugee/IDP Camp
			3 = School
			4 = Facility
			5 = Others
mWRD Availability	mWRD test available in LGA	Numeric	0 = No<br>1 = Yes in same community/area as ACF<br>2 = Yes, but in a different town/community in LGA<br>3 = Yes, coupled with Truenat or TB LAMP for ACF
TB diagnosis	Mode of confirmation of TB Diagnosis	Numeric	0 = Bact DS-TB case
			1 = Bact RR-TB Case
			2 = Clinical TB case

Diagnosed Date	Date Patient was confirmed as diagnosed with TB bacteriologically or Clinically (Date Test or CXR was reported)	Numeric	dd/mm/yyyy
Treatment Started	Has patient commenced TB Treatment after diagnosis	Numeric	0 = no<br>Yes = 1
Treatment Start Date	Date Patient was confirmed to have started treatment in the facility	Numeric	dd/mm/yyyy
Cough	Indicates presentation at screening with symptom of cough	Numeric	0 = no cough
			1 = Cough of <2 weeks duration
			2 = Cough of >2 weeks duration
Night Sweat	Indicates presentation at screening with symptom of Night sweat	Numeric	0 = No Night Sweat
			1 = Night sweat present
			2 = Unknown
Weight loss	Indicates presentation at screening with symptom of Weight loss	Numeric	0 = No Weight loss
			1 = Weight loss present

			2 = Unknown
Fever	Indicates presentation at screening with symptom of fever	Numeric	0 = No fever
			1 = Fever present<br><br>2 = Unknown
Previous_tb	Indicates presentation at screening with history of previous diagnosis/treatment for TB	Numeric	0 = no
			1 = yes
			2 = unknown/not disclosed
HIV	Indicates HIV positivity in patient based on test	Numeric	0 = no
			1 = yes
			2 = unknown/not disclosed
